# Healthy mitochondria inhibit the metastatic melanoma in lungs

**DOI:** 10.7150/ijbs.38104

**Published:** 2019-10-15

**Authors:** Ailing Fu, Yixue Hou, Zhenyao Yu, Zizhen Zhao, Zesheng Liu

**Affiliations:** College of Pharmaceutical Sciences, Southwest University, Chongqing 400715, China.

**Keywords:** isolated mitochondria, melanoma, bioenergy, redox

## Abstract

Tumor mitochondria alter their functions to reprogram cell metabolism and then allow tumor cells to rapidly proliferate in the hypoxic and acidic microenvironment. However, roles of normal mitochondria played in tumor progression are still unclear. Here we investigate the normal mitochondrial effect on abnormal metabolism of tumors, and to clarify why the mitochondria have to undergo functional changes in the tumor growth. The mitochondria isolated from healthy mouse livers were intravenously injected into melanoma model mice with lung metastasis, then the tumor growth, animal survival and associated metabolic changes were studied. The results reveal that the mitochondria significantly retard tumor growth and increase survival days of animals. The anti-tumor effect of the mitochondria is related to interfering the tumor cell metabolisms, such as reducing glycolysis and producing an oxidative intracellular environment, all of which are not suitable for tumor cell proliferation. In addition, the mitochondria increases cell apoptosis, necrosis, and mitophagy. These effects are more efficient with the mitochondria isolated from young mouse livers than those from aged mice. Our study not only provides a valuable approach to invest mitochondrial function associated with tumor growth but also offer new insight into tumor therapy through interfering the tumor cell metabolism by healthy mitochondria.

## Introduction

A major difference between tumor and normal cells is the tumor cell metabolic reprogramming, where tumor mitochondria play a crucial role in the reprogramming. Accumulating evidence identifies that tumor mitochondria change their structure and function since tumor initiation, and the abnormal mitochondria subsequently accelerate the tumor progression, which caused by decreasing the abilities of oxidative phosphorylation and inducing apoptosis 1,2,3 However, the intrinsic reason for the alteration of mitochondrial function is still unclear, and the action of healthy mitochondria in tumor proliferation is also under-investigation.

Delivery of healthy mitochondria into tumor cells would be a most direct and effective approach to clarify the roles of the mitochondria played in the cells. In our previous studies, we indicate that systemic injection of isolated mitochondria can reduce liver injury induced by acetaminophen and high-fat diet through improving hepatocyte energy supply and increasing cell viability.^4^,^5^ The exogenous mitochondria could be developed as a micro-sized agent to increase cell survival since the mitochondria can maintain their morphology and function in recipient cells.^6^, ^7^

**Figure 1:**
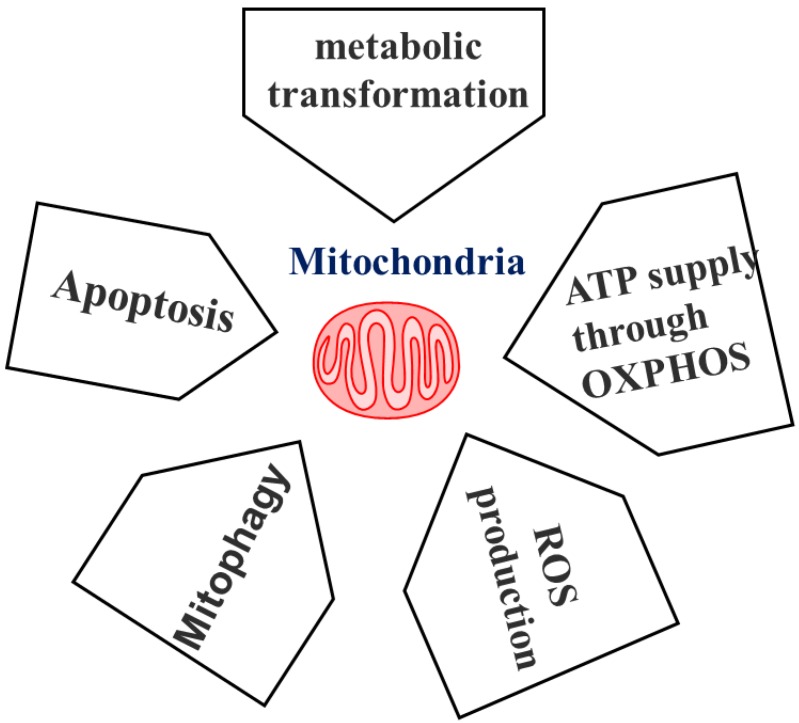
Main functions of mitochondria.

Here, we describe the transfer of healthy mitochondria into melanoma-bearing mice to evaluate the effect of the mitochondria in the tumor cells, and identify the mechanisms that are closely associated with main functions of mitochondria (Fig. 1). Melanoma is the most lethal form of skin cancer and its incidence is rapidly rising in recent years, and the major mortality of the melanoma is tumor metastasis.^8^ In the study, we use the mouse model of melanoma lung metastasis to determine the survival days of the melanoma-bearing mice after mitochondrial administration. Also, two sources of mitochondria respectively isolated from young and old mouse livers are compared to confirm the effect of healthy mitochondria. The study would not only clarify the reason that tumor mitochondria have to undergo functional changes in tumor metabolism, but also provide a cancer-specific therapeutic strategy.

## Results

### Characteristics of isolated mitochondria from young and aged mice

**Figure 2:**
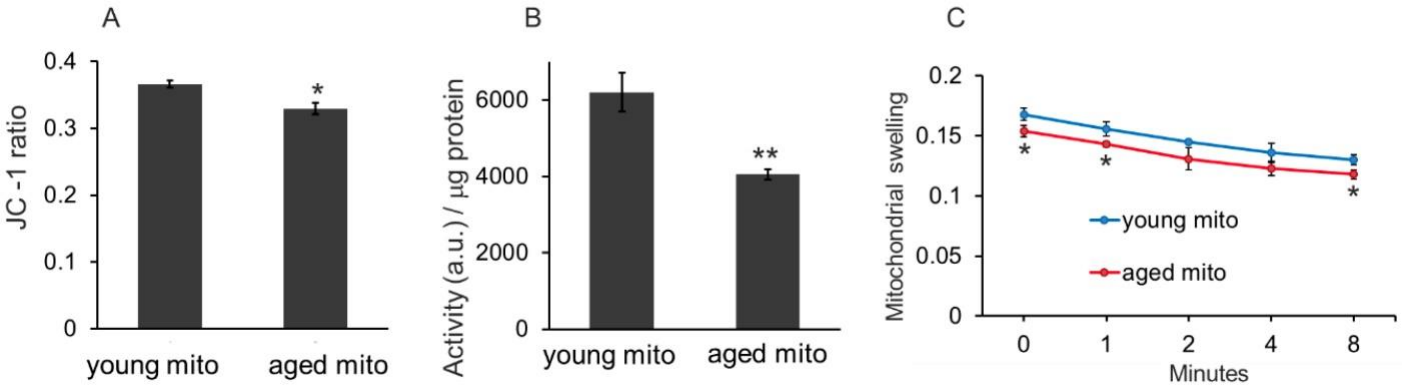
** The difference between mitochondria isolated from young and aged mice. (A),** the membrane potential was assayed by JC-1 method**. (B),** mitochondria redox ability was measured as the ratio of fluorescence intensity (a.u./mL) to mitochondrial suspension concentration (μg/mL). **(C),** mitochondrial swelling test. Young mitochondria mean mitochondria from young mice, and aged mitochondria indicate mitochondria from old mice. Mito, mitochondria. *p < 0.05, **p < 0.01 compared with the young mito. Student's t test was employed to compare the difference between the young and aged mito groups.

Three methods, mitochondrial membrane potential, redox ability, and mitochondrial swelling tests, were used to examine the difference between the mitochondria isolated from young and aged mice. The mitochondrial membrane potential is detected by JC-1 assay, which is based on the principle that when the mitochondrial membrane remains intact, intense red fluorescence of JC-1 aggregates will be detected in the isolated mitochondria, but when the mitochondrial membrane is disrupted, the membrane potential will reduce, then green fluorescence of JC-1 monomers can be detected. In the study, the ratio of red and green fluorescence intensity of young mitochondria was higher than that of the aged mitochondria (Fig. 2A). Similarly, the redox ability of young mitochondria was significantly higher than that of aged mitochondria (Fig. 2B). Moreover, the mitochondrial swelling test indicated that absorbance of the aged mitochondria relatively decreased compared with that of the young mitochondria (Fig. 2C). These results suggested that oxidative capability of aged mitochondria reduced, which is consistent with the previous studies.^9^,^10^

### Distribution of mitochondria in melanoma

**Figure 3:**
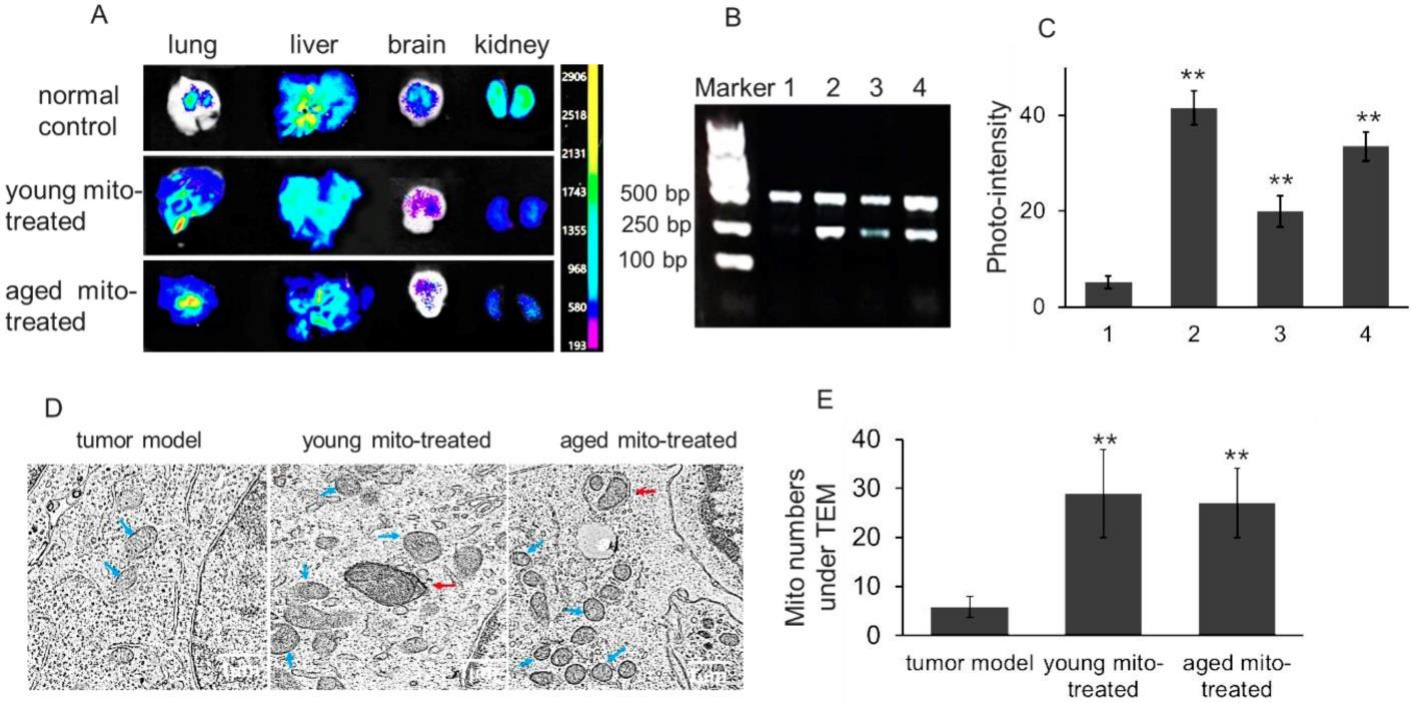
** Isolated mitochondria arrived in melanoma cells of lung metastasis. (A),** tissue fluorescence after mitochondrial administration. The mitochondria were pre-stained by picogreen, a specific dsDNA indicator. The tissues were dissected out at 4 h after mitochondrial injection (10^8^ mitochondria). **(B),** competitive PCR was used to detect aged mitochondria (containing deletion mtDNA) entry the melanoma cells, and then the photo-density was quantified **(C).** 1, melanoma; 2, aged mitochondria; 3, melanoma of the mice that received once mitochondrial administration; 4, melanoma of the mice that received repeatedly mitochondrial injection. **(D),** the melanoma cells of lung metastasis was observed under TEM. Blue arrows point to intracellular mitochondria, and red arrows to mitophagy. **(E),** quantification of mitochondrial counts in the TEM images. The intracellular mitochondrial numbers increased after the mice were administrated with isolated mitochondria. The data are expressed as the mean ± SD (n = 15 per group). **p < 0.01 compared with tumor model group.

To determine the distribution of isolated mitochondria in melanoma after intravenous injection, the lung, liver, brain, and kidney were respectively dissected out after the melanoma-bearing mice received picogreen-labeled mitochondria. PicoGreen is a specific dsDNA fluorescence indicator that associates to DNA as an intercalator and minor-groove binder.^11^ The result showed that stronger fluorescence appeared in the melanoma-metastasizing lung than the normal lung of mice (Fig. 3A), which might be associated with the enhanced permeability and retention (EPR) effect of tumor tissue.^12^ In addition, melanoma mtDNA was extracted after aged mitochondria administration for competitive PCR reaction by using primers F1, F2 and R simultaneously, and the PCR products were subjected to 2% agarose gel electrophoresis. Under UV, all PCR products showed two bands that appeared at 469 bp and 256 bp, in which the 469 bp band represents as wild type mtDNA, while the 256 bp band as deletion mtDNA. The content of deletion mtDNA increased in aged mouse tissues, including brain, lung, and liver.^13^,^14^ The image showed that 256 bp band was very weak in melanoma, while high photo-density appeared in the aged mitochondria (Fig. 3B and 3C). Meanwhile, the photo-density of 256 bp band increased in melanoma when the aged mitochondria were injected to the mice, and relative content reached 66.5% after the mice received repeatedly mitochondrial administration (Fig. 3C). To further examine the mitochondrial content after injection, TEM was used to observe approximate numbers of intracellular mitochondria. The images exhibited increasing mitochondrial numbers following aged and young mitochondria administration (Fig. 3D and 3E). These results identified that isolated mitochondria could enter metastatic lung melanoma.

### Mitochondria inhibit melanoma lung metastasis and increase animal survival

**Figure 4:**
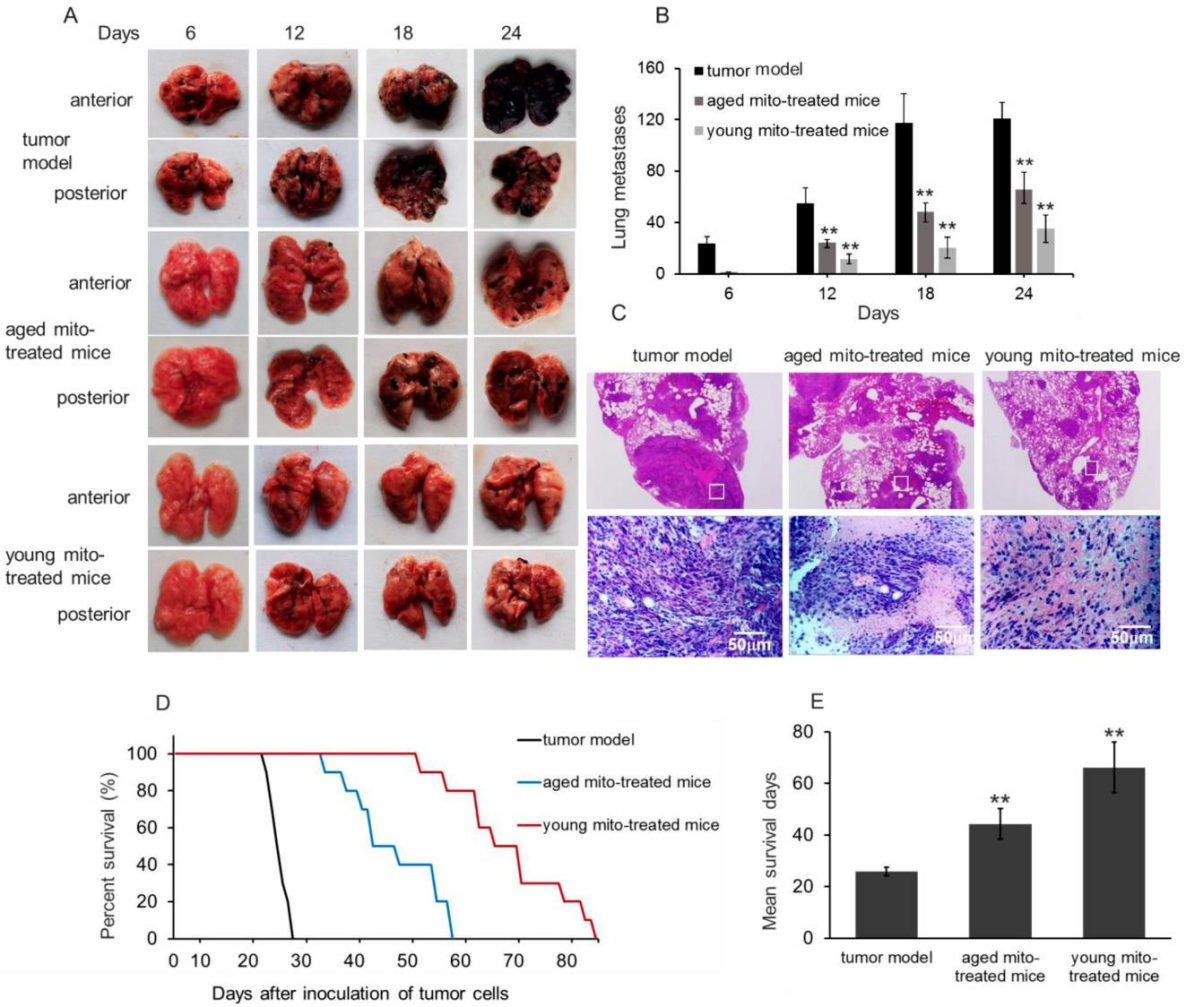
** Mitochondria inhibited tumor metastasis and prolong survival. (A),** effects of mitochondria on melanoma lung metastasis, visible as black nodules on the surface of lung lobes from mice treated by PBS (tumor model), aged and young mitochondria. **(B),** total number of melanoma lung metasitatic colonies observed in the tumor model, aged and young mitochondrial group. **(C),** HE staining showed the nodules of melanoma lung metastatic colonies. The upper pictures were magnified 10. The images in white squares were zoomed in 200 times (below pictures). **(D),** survival curve and **(E),** mean survival days of three group of tumor-bearing animals. All data are expressed as the mean ± SD (n = 10 ~ 12 per group). ^##^* p* < 0.05 compared to normal control, and *p < 0.05, **p < 0.01 with model group.

After melanoma cell transplantation, the numbers of lung melanoma nodules gradually increased and the lung color was getting dark (Fig. 4A and 4B). The control mice at autopsy showed large and dense nodules on day 24, evaluated by gross anatomy and HE staining (Fig. 4C). However, in mitochondria-treated mice, tumor formation and progress were obviously retarded, and the numbers of metastatic melanomas per lung in the mitochondria-treated group were significantly lower than that in the control group (Fig. 4B). Also, the numbers of melanomas per lung in the young mitochondria group were lower than that in the aged mitochondria group (Fig. 4B). Moreover, the nodules became small in the mitochondria group, and especially, the nodules appeared smaller and scattered in the young mitochondria-treated mice compared to the aged mitochondria-treated littermates at the day 24 after the cell transplantation (Fig. 4C).

Survival study showed that melanoma-bearing mice naturally died between 24 and 28 days. However, mitochondrial treatment with repeat injection of once in two days resulted in an appreciable prolongation of the survival days (Fig. 4D and 4E). Administration of mitochondria led to a mean survival day of about 44 days (aged mitochondrial group) and 66 days (young mitochondrial group), respectively. Of these mice, several mice died before the mean survival days (53, 58, 59 days) in the young mitochondria group, but the lungs were melanoma a few and other organs melanoma free, despite the reason for death remains uncertain.

The results clearly show that the mitochondria could inhibit tumor growth and prolong the survival significantly, especially with young mitochondria, indicating that the young mitochondria with normal function could effectively inhibit cell proliferation. This is the first report in mitochondrial inhibition of tumor *in vivo*.

### Mitochondria could regulate glucose metabolism and glutaminolysis to inhibit tumor growth

**Figure 5:**
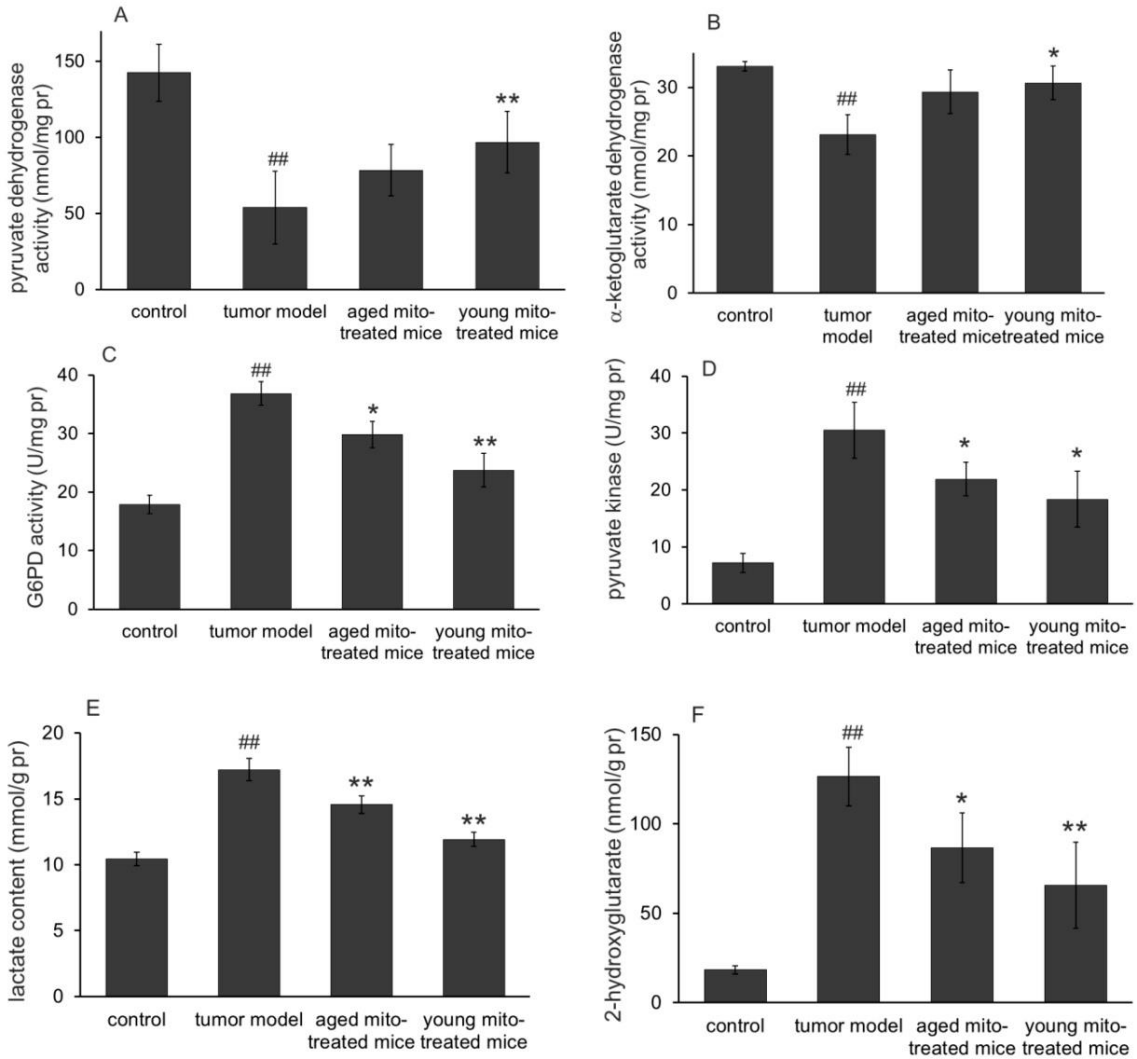
** Effects of the mitochondria on enzymes and metabolites of glucose metabolism.** The activities of pyruvate dehydrogenase **(A)** and α-ketoglutarate dehydrogenase **(B)** in melanoma increased after mitochondrial administration, while glucose-6-phosphate dehydrogenase **(C)** and pyruvate kinase activities **(D)** decreased. As a result, the lactate content reduced when the mitochondria prevented the tumor glycolysis. Moreover, 2-hydroglutarate (a tumor metabolic indicator) level in melanoma decreased after mitochondrial therapy. ^##^* p* < 0.05 compared to normal control, and *p < 0.05, **p < 0.01 with tumor model group.

**Figure 6:**
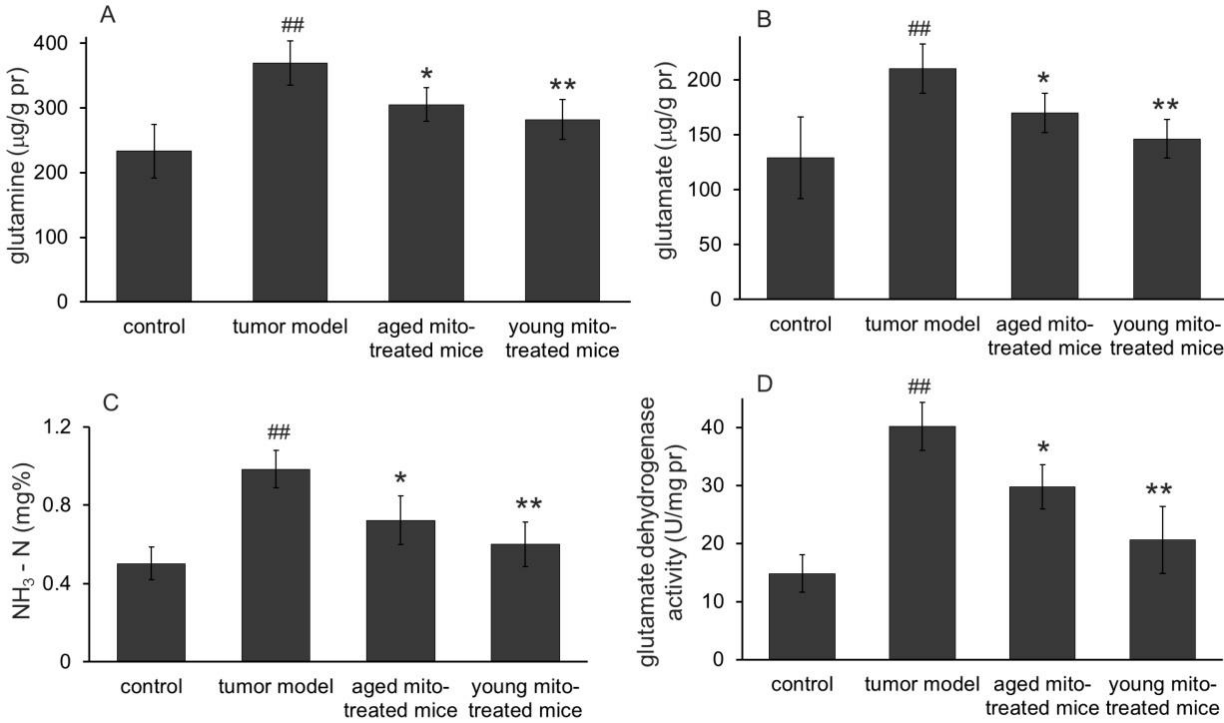
** Effects of the mitochondria on glutaminolysis.** The mitochondria reduced the levels of glutamine **(A),** glutamate **(B),** and ammonia **(C)** in melanoma. Also, the mitochondria down-regulated glutaminolysis by decreasing the activity of glutamate dehydrogenase (D). ^##^* p* < 0.05 compared with the normal control, and *p < 0.05, **p < 0.01 with the tumor model group.

One of the main functions of mitochondria is involved in substance and energy metabolism. Tumor mitochondria have reprogramed the metabolism pathways to maintain their rapid reproduction, where altered glucose metabolism (the Warburg effect) and increased glutaminolysis are known the two typical features in proliferative tumor cells. Here we investigate whether the healthy mitochondria inhibit the tumor growth through interference the abnormal metabolic pathways of the tumor mitochondria. The results were shown in Fig. 5 (glucose metabolism) and Fig. 6 (glutaminolysis). The results clearly indicated the dominant glycolysis in metastatic melanoma cells with increased lactate production through inhibiting the activity of pyruvate dehydrogenase (PDH) and α-ketoglutarate dehydrogenase (αKDH), while increasing glucose-6-phosphate dehydrogenase (G6PD), pyruvate kinase (PK) activity (Fig. 5). Moreover, healthy mitochondria could reduce the content of 2-hydroxyglutarate, an oncometabolite that is dramatically increased in tumor group, which comes from mutated isocitrate dehydrogenase (IDH) in tumors. Meanwhile, the increased glutamine, glutamate, and ammonia levels (Fig. 6) were detected out in tumor group, suggesting the altered metabolic pathways with high glycolysis and glutaminolysis. However, the healthy mitochondria could significantly increase the activity of PDH and αKDH, and meanwhile decrease the activity of G6PD and PK (Fig. 5). Importantly, healthy mitochondria could also down-regulate glutaminolysis by reducing the activity of glutamate dehydrogenase (GDH) and contents of glutamine, glutamate and ammonia (Fig. 6), which could be a synergistic effect to stop tumor growth.

### Effect of the mitochondria on oxidative stress and ATP production

**Figure 7:**
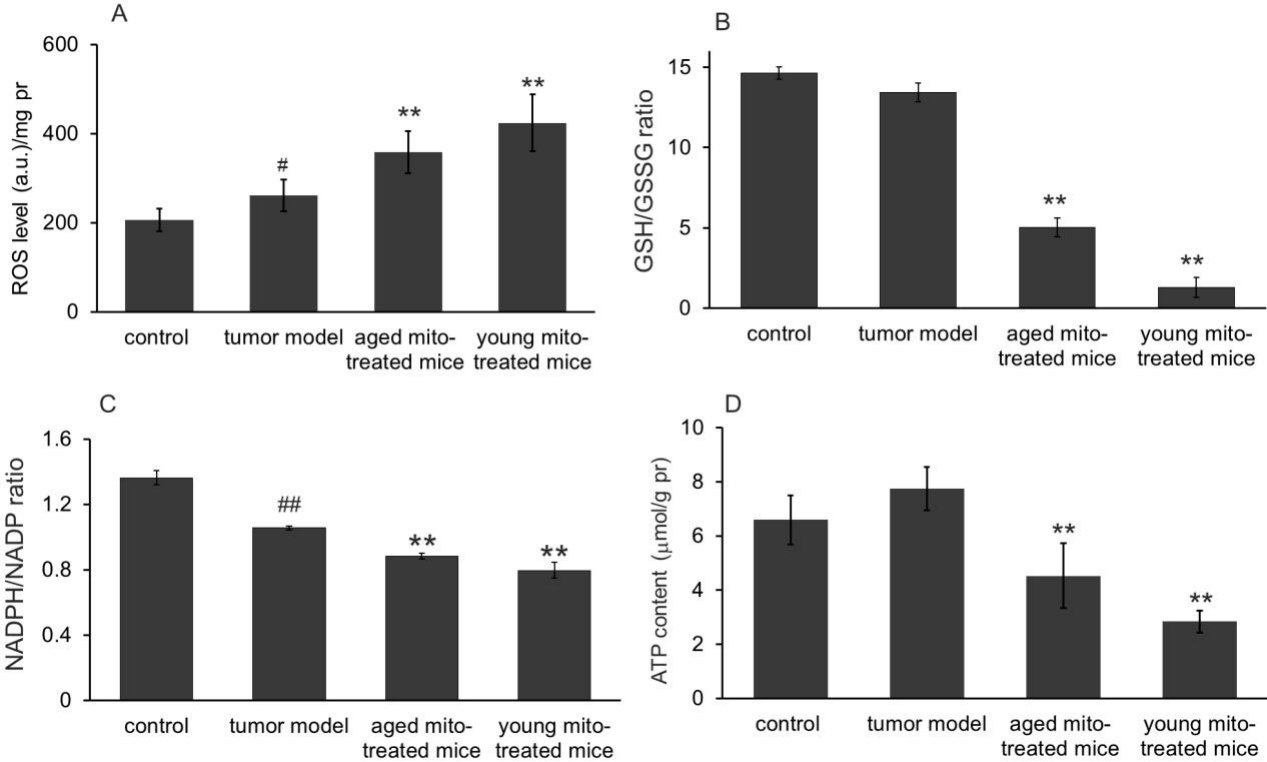
** Effects of mitochondria on bioredox and bioenergy. (A),** ROS level increased in tumor cells compared to control, however, increased ROS in both mitochondrial treatment, especially in young group. **(B),** mitochondrial treatment significantly reduced GSH/GSSG ratio in both group. **(C),** the reduced ratio of NADPH/NADP^+^ in tumor cells were further decreased by mitochondria in both groups. (D), slightly raised ATP content in tumor cells significantly reduced, especially in young mitochondria-treated group. ^##^* p* < 0.05 compared to normal control, and *p < 0.05, **p < 0.01 with model group.

We further investigated whether the healthy mitochondrion with normal metabolic function would influence the tumor metabolisms of bioredox and bioenergy in the melanoma since mitochondria are responsible for ROS and energy production. As expected, melanoma cells showed an increased ROS level compared to the normoxic lung tissue (control) (Fig 7A). However, after 24 days of mitochondrial administration, ROS level increased 37% in aged mitochondria and 61.9% in young mitochondria compared with the tumor model mice (Fig 7A). Correspondingly, the level of intracellular anti-oxidant agent GSH decreased, while GSSG increased dramatically, leading to the dropping of GSH/GSSG (Fig 7B), an indicator of reducing status within the cell. Another pair of reducing indicator in cells is the ratio of NADPH/NADP. Thus its ratio was also measured in melanoma that treated with mitochondria. The results showed that this ratio in tumor cells significantly reduced compared with the control, indicating the unfavorable environment in tumor tissue, probably due to increased ROS (Fig 7A). Decreases of the GSH/GSSG and NADPH/NADP ratio were seen in both aged and young mitochondria-treated groups (Fig 7C), indicating that it is the oxidative status but not reducing environment induced in the tumor after mitochondrial administration. These results demonstrated that healthy mitochondria could change redox condition in the tumor, by producing an oxidative status that is not favorable for cell proliferation.

The ATP contents in melanoma homogenate were also tested. The result showed that ATP content in melanoma was slightly increased compared with the control (Fig 7D), which suggests that the tumors have strong glycolysis with increased lactate content. However, after mitochondrial administration, The ATP content was dramatically reduced in both mitochondria-treated groups, especially in the young mitochondria-treated group (Fig 7D). It clearly indicates that the mitochondria could rapidly obstruct ATP production from glycolysis, leading to energy starvation of the tumors.

### Mitochondria could increase p53 level

**Figure 8:**
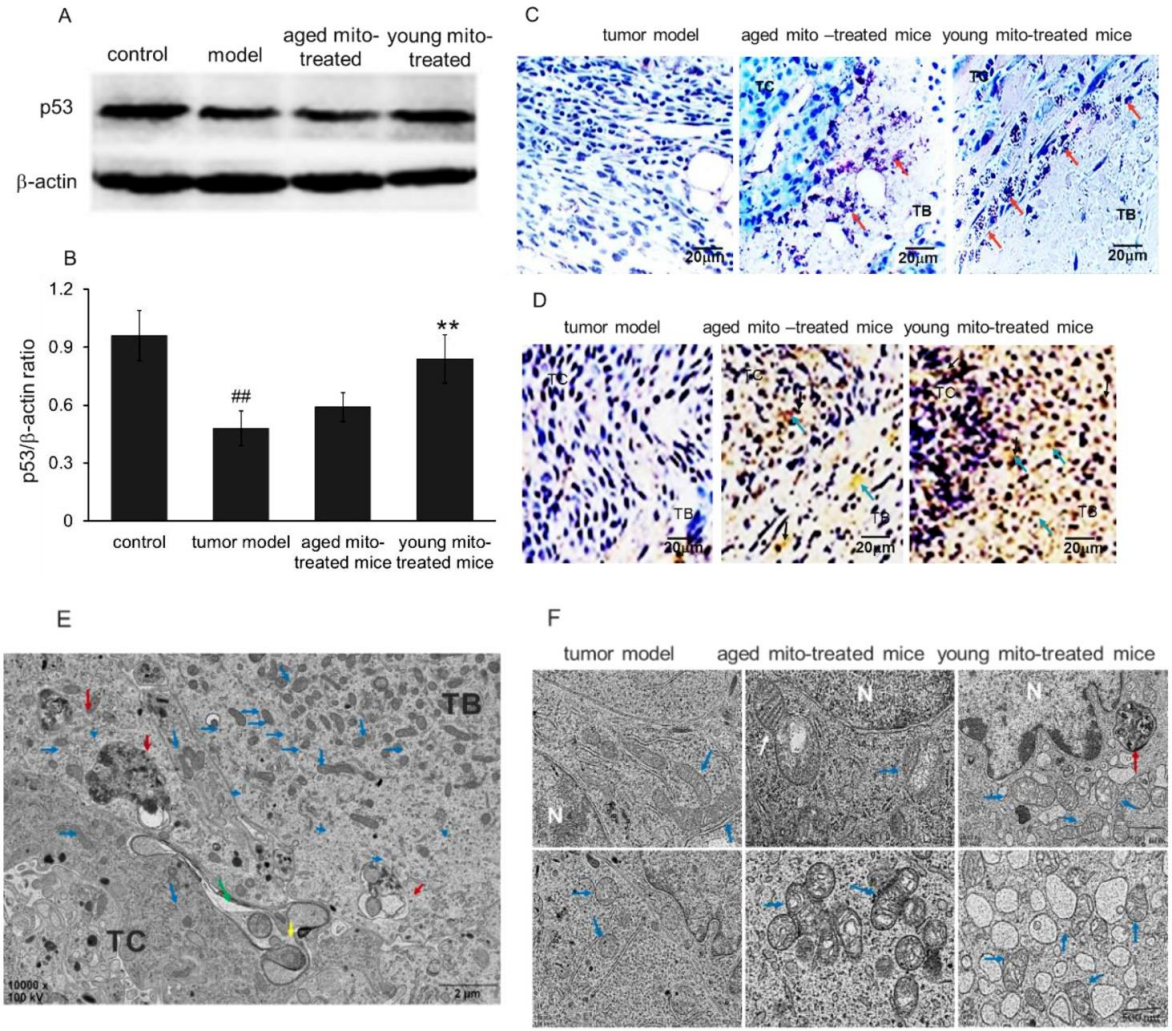
** Mitochondria induced cell death via apoptosis and necrosis pathways. (A),** western blot analysis of p53 protein expression in tumor model, and treated with aged and young mitochondria. **(B),** the qualitative results of western blot analysis demonstrated that p53 expression was reduced in tumor model, but increased almost to the control level by young mitochondria reatment **p < 0.01. In addition, cell apoptosis was measured by POD **(C)** and TUNEL staining (× 100) **(D).** TC, tumor central; TB, tumor boundary. The TUNEL-positive cells were developed by DAB (brown color). The nuclei were stained by hematoxylin. The black arrows point to apoptotic cells. **(E),** TEM observation of mitochondria in melanoma cells after mitochondrial administration. Low magnification images of TEM showed the overall morphology of mitochondria and cells. Mitochondria mainly concentrated in TB and diffused to TC, and several macroautophagosome appeared in the cells. **(F),** cell morphology and mitochondria in the three groups of tumor model, aged and young mitochondria. Blue arrows point to intracellular mitochondria, the yellow to mitochondria at the cell boundary, and the green to mitochondria in intercellular spaces. The red arrows point to autophagosomes, and the white to fission mitochondria.

p53 plays vital role in monitoring redox milieu and energy production pathway to inhibit cell survival in the oxidative environment and prevent cell respiration through fermentation. p53 activation is cross-linked with ROS status due to its redox sensor, cysteine residues, which can detect ROS level by oxidized into S-S bond and then induce p53 conformational change, leading to trigger cell death in the high level of ROS.^15^,^16^ Therefore, it is possible that healthy mitochondria could associate or influence p53 level in tumors. Bearing this in mind, we investigated p53 level by Western blot after the mitochondrial administration. Melanoma homogenates from the control, tumor model and both mitochondria-treated groups were analyzed with β-actin as an internal control (Fig 8A). As showed in Fig 8B, p53 expression was reduced into half of the amount in tumor tissue compared with the control, and a slightly increased of p53 level in the aged mitochondrial group compared with tumor group, but a remarkable increasing in young mitochondria group (Fig 8B), suggesting that young mitochondria have the ability to recover the p53 level while aged mitochondria with functional deficiency could not increase the level of p53 back to normal.

### Mitochondria triggers cancer cell death through apoptosis and necrosis

p53 and/or ROS can trigger cell apoptosis by the mitochondrial pathway. Thus, TUNEL staining was performed to identify apoptosis after mitochondrial administration. As shown in Fig 8C and 8D, exogenous mitochondria increased apoptosis of the tumor cells. The liquefaction area and junction zone exhibited the amount of apoptotic body and damaged cells (Fig. 8C), whose nuclei were deeply stained with agglomerate chromatin. Fig 8D showed that numbers of TUNEL-positive cells were stained brown by DAB after mitochondrial administration. Particularly, the tumor cells treated with young mitochondria appeared more TUNEL-positive cells than those of aged mitochondria.

Moreover, the melanoma nodules were dissected out and the cells were observed under the TEM. The image showed that large numbers of mitochondria appeared in the tumor border (TB), and were migrating to tumor central (TC) after mitochondrial administration (Fig. 8E). Also, the melanoma of tumor-bearing mice treated with aged mitochondria exhibited typical mitochondrial fission, aggregation, and autophagic vacuoles, but no or few aggregations and autophagic vacuoles in the control cells of tumor model mice (Fig. 8E, 8F, and 3D). The melanoma-bearing mice with young mitochondria treatment showed more obvious cytotoxicity in the tumor cells than that of aged mitochondria, including damaged mitochondria, numerous autophagic vesicles, endoplasmic reticulum swelling, ribosome abscission and nuclear deformation (Fig. 8F). These results indicate that mitochondria treatment induces cellular metabolic disturbance and even necrosis in melanoma cells.

## Discussion

Mitochondria are not only cells' powerhouse that is indispensable for cell survival but also cells' suicidal weapon store that are crucial regulators of cell death. The role of mitochondria in regulating cell survival or death depends on cell states. Here, we uncover that healthy mitochondria change the milieu that adapts proliferating tumor cells to the state of oxidative stress and energy depletion, which consequently leads to tumor cell death (apoptosis and necrosis). Moreover, the effect of tumor inhibition in oxidative status and energy depletion is more profound in the young mitochondria-treated group compared to aged mitochondria-treated mice.

Isolated healthy mitochondria have been reported to provide energy to somatic cells, and increase cell survival and growth.^17^,^18^ However, here we show that isolated mitochondria inhibit tumor cell proliferation and induce cell death. The distinct effect lies in the different roles of mitochondria played between tumor and somatic cells. Mitochondrial structure and function have obviously altered in tumors, and the abnormal mitochondria involve in metabolic reprogramming to adapt unrestrained proliferation of tumor cells. One of the characteristics of tumor mitochondria is to provide metabolic intermediates through truncated or reversed TCA cycle using certain nutrients (such as glutamine). Moreover, the abnormal mitochondria shift tumor cell respiration from the oxidative phosphorylation to aerobic glycolysis, which is referred to the initiating step in tumorigenesis.^19^ In addition, the numbers of mitochondria in tumor cells also changed that malignant tumor cells have fewer mitochondria numbers than somatic cells.^20^,^21^,^22^ The decreased mitochondrial numbers reduce oxygen consumption to adapt the tumor hypoxic microenvironment that is induced by highly proliferative tumor cells, distancing themselves from an oxygen supply and avoiding cell apoptosis dominated by mitochondria in hypoxic conditions.^23^ Similarly to other tumors, mitochondrial functions in melanoma cells are altered, and the cells exhibit increased glycolysis and decreased oxidative phosphorylation, resulting in obviously increasing of lactate content.^24^ Then the rapidly growing melanoma aggravate tissue hypoxia that further induces the proliferation and metastasis of the tumor with anaerobic fermentation.^25^ Moreover, The healthy mitochondria might cross the vascular endothelial cells through transcytosis pathway, by which particles overpass the wall of blood vessels.^26^ Then the mitochondria may enter the tumor cells by endocytosis, which is suggested the entry mechanism of the exogenous mitochondria into cells.^27^

**Figure 9:**
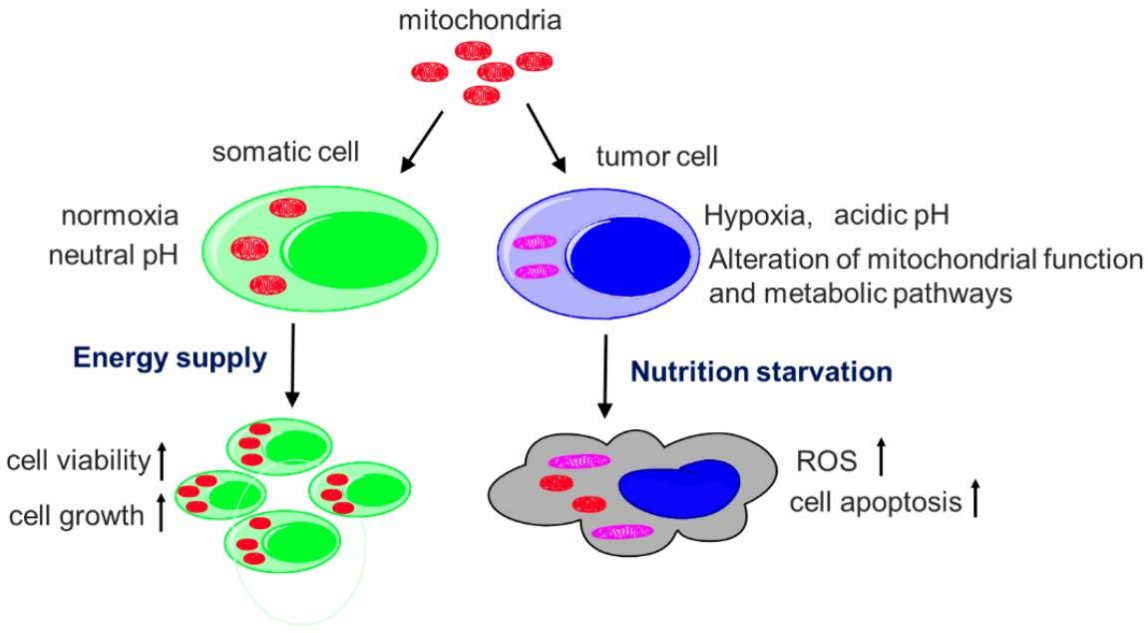
** Diagram of the mechanisms of selective toxicity to melanoma cells.** Hypoxia and acidic pH microenvironment are two typical hallmarks of tumor that is strongly associated with invasion, metastasis, resistance to therapy and poor clinical outcome. Tumor mitochondria alter their structure and function to adapt the microenvironment. However, isolated mitochondria that still sense the oxygen change, cannot bear the hostile microenvironment and then produce cytotoxic to the tumor. The apoptosis is induced when the isolated mitochondria enter tumor cells. However, the somatic cells improve the viability after they receive the mitochondria.

Since structural and functional alterations of tumor mitochondria are common features in tumors, it undoubtedly provides a unique opportunity to develop reagents to target tumor.^28^,^29^ When insolated normal mitochondria that depend on oxygen enter anoxic tumor cells, they will rapidly cause oxygen depletion and nutrition starvation, inducing ROS production, apoptosis, and even cell necrosis (Fig. 9). Also, in the acidic extracellular microenvironment of tumor cells, protons pass through the proton channels of intact mitochondrial membrane, which will drive oxygen-consuming and ROS generation. As a consequence, the exogenous mitochondria inhibit tumor cell proliferation when they enter the cells.^30^ On the contrary, when somatic cells that depend on mitochondria as ATP producer, are supplemented by isolated mitochondria, cell viability would increase and injured cells could have sufficient energy to recover their function. The selective cytotoxicity to tumor cells would make mitochondria a promising anti-tumor candidate.

**Figure 10:**
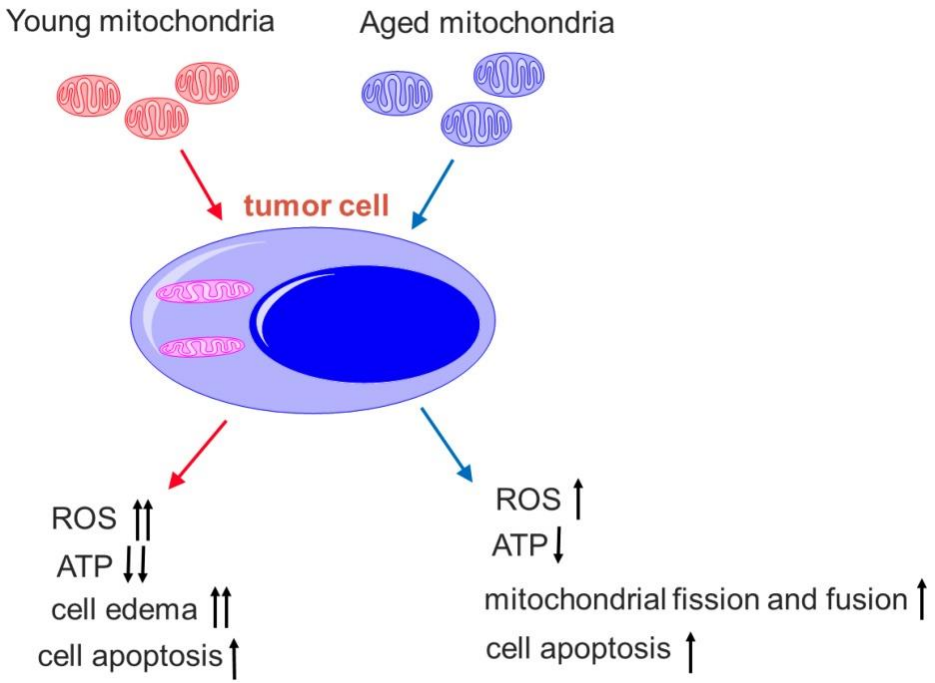
** Comparison of young and aged mitochondria on the tumor treatment.** The young mitochondria are more dependent on oxygen than aged mitochondria. Once in the microenvironment of hypoxia and nutrition starvation, they produce higher toxic to the tumor cells than the aged mitochondria.

Here, we also find that the mitochondria from young healthy mice have higher efficacy to inhibit tumor cell proliferation than the mitochondria from aged mice. There are mtDNA mutations in aged animals, and mitochondrial ability to produce ATP reduces accordingly,^31^ then the oxygen dependence and consumption of aged mitochondria are lower than the mitochondria from young mice. Thus, the toxicity of mitochondria from aged mice on tumor cells is relatively lower than that of mitochondria from young mice under the hypoxic microenvironment of the tumor (Fig. 10). Nevertheless, mitochondria from aged mice also promote mitophagy and tumor cell apoptosis. In addition, tumor cells try to eliminate damaged parts of the aged mitochondria through fission and fusion (Fig. 8B), which is not observed in the tumor treated by the mitochondria from young mice.

Moreover, the study showed an increased tumor suppressor p53 level after mitochondrial administration. It's reported that increased p53 triggers cell death that can be through apoptosis and necrosis, and mitochondria involve in all of the pathways.^32^ Meanwhile, increased p53 can regulate necrotic cell death via mitochondria, in which p53 accumulates in the inner mitochondrial membrane to associate with cyclophilin D (CypD) to form complexes during bioenergetics failure, then promotes MPTP opening and mitochondria-related necrosis. In our results of young mitochondrial administration in melanoma, a continuum of apoptosis and necrosis exists at the time of p53 and ROS increase. It's known that apoptosis appears at lower oxidative stress state and necrosis at higher state, and the mitochondrial distribution in melanoma after mitochondrial administration is uneven with more mitochondria in tumor boundary and less in the core, then it causes a coexisting scenario with a necrotic penumbra surrounding an apoptotic center.

In summary, our results provide new insight into mitochondrial action in tumor, and propose that healthy mitochondria inhibit tumor cell proliferation by alteration of tumor metabolisms. Tumor mitochondria would have to alter their function to adapt the tumor growth because healthy mitochondria damage the cells with ROS attack and energy depletion. Moreover, the effect of the mitochondria on melanoma would make it a potentially promising therapy in other malignant tumors.

## Materials and Methods

### Animals

Healthy male BABL/c mice with the age of 2 months (young mice) and 18 months (aged mice), were used in the study. The mice were obtained from Chongqing Medical University, Chongqing, China. The animal experiments were performed following the guidelines and approved by the Animal Committee of Southwest University, China.

### Cell culture and preparation of melanoma lung metastasis model

Murine melanoma B16F10 cells were a gift from Dr. Lei Luo with the American Type Culture Collection (ATCC; Manassas, VA, USA) as the original source. These cells were cultured in RPMI-1640 medium supplemented with 10% fetal bovine serum (Gibco, Carlsbad, CA, USA), 100 units/mL penicillin, 100 μg/mL streptomycin, and incubated in a 37°C incubator (CCL-170B-8, ESCO, Timur, Indonesia) containing 5% CO_2_.

To prepare the tumor model, young mice were anesthetized with 3% sodium pentobarbital, and then intravenously injected with 1 × 10^6^ B16F10 cells diluted in 200 μL PBS via the tail vein. At 6, 12, 18, 24 days after injection, the mice were euthanized by overdose sodium pentobarbital, and numbers of metastatic colonies per lung were assessed.

### Isolation of mitochondria from mouse liver

Liver mitochondria were isolated according to the previous reports.^33^,^34^ Briefly, mice were euthanized by cervical dislocation, and then the liver was dissected out immediately. The liver was washed by cold PBS and cut into pieces. Subsequently, the samples were homogenized in cold isolation buffer, then the homogenate was centrifuged at 800 g for 5 min at 0 ~ 4°C. The supernatant was re-suspended in the isolation buffer for another centrifugation for 10 min. The mitochondrial was washed with the isolation buffer and placed at 4°C before use. The mitochondrial concentration was detected by BCA assay.

### Characteristic of the isolated mitochondria

Mitochondrial membrane potential was determined by JC-1 assay kits (Jiangsu Kaiji Biotech. Ltd. Co, Nanjing, China), and fluorescence spectrophotometer (F-7000; Hitachi, Japan) was used to measure the red (Ex/Em 525/590 nm) and green (Ex/Em 490/530 nm) fluorescence intensity. The ratio of red and green fluorescence intensity was calculated. The measurement was independently repeated for three times.

Mitochondrial redox ability was assayed by resazurin method.^35^ Briefly, mitochondrial suspension (50 μg/mL) was added into a 96-well plate, then 0.5 mmol/L resazurin solution was added to the final concentration of 5 μmol/L. The mixture was incubated at 37°C for 30 min. The fluorescence intensity (Ex/Em 530/590 nm) was detected under the fluorescence spectrophotometer.

Mitochondria swelling were measued by CaCl_2_-induced method according to previous report 4. Briefly, mitochondrial suspension was added into the homoosmotic buffer for incubation, then 6.5 mmol/L CaCl_2_ were added in room temperature. The absorbance (550 nm) was recorded at 0, 1, 2, 4, 8 min respectively following CaCl_2_ addition.

### Distribution of the isolated mitochondria in tumor

Mitochondrial distribution was analyzed according to the previous reports 4,22. Briefly, transmission electron microscope (TEM), fluorescence imaging and competitive PCR analysis were used to examine the distribution of mitochondria in melanoma tumor cells.

Transmission electron microscope (TEM) observing, fluorescence imaging, and competitive PCR analysis were used to examine the distribution of mitochondria in the melanoma. The melanoma-bearing mice (21 days after B16F10 cell transplantation) were intravenously administrated mitochondrial suspension (10^8^ mitochondria). After 4 h injection, the tumor was dissected out for TEM (H7500, Hitachi Ltd) observing according to the operation manual.

Fluorescence imaging *in vivo* was carried out by using an *In vivo* Imaging System FX Pro (Carestream Health Inc., Rochester, NY, USA). The mitochondria were pre-stained by picogreen (a dsDNA fluorescent indicator). After the melanoma-bearing mice received mitochondrial injection, the mice were deeply anesthetized with overdose sodium pentobarbital, and then transcardically perfused with cold PBS (0.01 M, pH 7.4) to remove blood. Mouse lung, liver, brain, kidney was respectively excised and placed in the cassette. Images were captured with optical and fluorescence (Ex/Em 470/535 nm). The images were overlaid according to the protocol of the manufacturer.

For competitive PCR analysis, melanoma nodules at mouse lung were removed and homogenized in cold PBS. Then mitochondrial DNA (mtDNA) was extracted as previously described.^36^ Three primers, F1 (5' TCTATTCATCGTCTCGGAAG 3'), F2 (5' TACCATTC CTAACAGGGTTC 3') and R (5' TTTATGGGTGTAATGCGGTG 3'), were synthesized. Primer F1/R was used to amplify deletion mtDNA fragment, while F2/R to wild mtDNA sequence.^37^ All of the three primers were added to the PCR reaction system. Then the PCR products were subjected to 2% agarose electrophoresis in TAE buffer (0.04 M Tris-acetate, 0.001 M EDTA, pH 8.0) at 80 mV for 10 min. The DNA bands were observed and quantified under Gel Doc XR (Bio-Rad, USA). The relative percentage of deletion mtDNA was represented as deletion mtDNA / (deletion mtDNA + wild mtDNA) × 100%.

### Assessment of metastatic melanoma of the lung

The mice, after being injected with melanoma cells, were randomly assigned into three groups (n = 10 ~ 12 for each group): tumor group and two mitochondria groups [young mitochondria group (mitochondria isolated from young mouse liver) and aged mitochondria group (mitochondria from aged mouse liver)]. Mice in mitochondria groups were respectively received young or aged mitochondrial injection (10^7^ mitochondria each time) via tail vein once in two days at the third days after the tumor inoculation. The mice in tumor group were injected with the same volume PBS in parallel. At 6, 12, 18, and 24 days after tumor inoculation, the numbers of metastatic colonies per lung was counted after anterior and posterior image acquisition.

### Tissue staining and biochemical assay

Mouse lung were quickly excised at 24 days after tumor inoculation and one part of lung tissue was fixed in buffered 10% formalin for tissue section for histology analysis. The remaining part was snap-frozen in liquid nitrogen for preparation of tissue homogenate for biochemical assay. The formalin-fixed lung tissue were imbedded in paraffin and cut into 5 μm thin slices. Then the tissue sections were stained with hematoxylin and eosin (HE).

Biochemical analysis on the levels of ROS, ATP, lactate, GSH/GSSG, NADPH/NADP, NH_3_-N, a-ketoglutarate, 2-hydroxyglutarate, glutamate, glutamine and enzyme activities of glutamate dehydrogenase, pyruvate kinase, α-ketoglutarate dehydrogenase, glucose-6-phosphate dehydrogenase and pyruvate dehydrogenase in melanoma homogenates were performed by using commercial kits (Nanjing Jiancheng Biotech. Ltd. Co., Nanjing, China and Shanghai Youxuan Biotech. Co. Shanghai, China), was according to the respected protocols. The ultrathin tumor sections of the melanoma nodules were used for mitochondrial observation under the TEM. Three independent experiments were performed for each assay.

### Western blotting

Western blotting was performed to determine whether the p53 level in lung melanoma changed after mitochondrial treatment. The mice were euthanatized at 24 days after the cell transplantation, and melanoma in the lung was excised and tissue samples were homogenized in ice-cold lysis according to the previous report.^38^ Briefly, samples (20 μg protein per lane) were subjected to SDS-PAGE gel electrophoresis and electrophoretically transferred to the PVDF membranes (Amersham Pharmacia, Piscataway, NJ, USA). The membranes were blocked in the blocking solution containing 5% skimmed milk at room temperature, and then immersed with the wild p53 mouse polyclonal antibody (Beijing Saibaisheng Biotech. Co., Beijing, China; 1:1000). Membranes were washed twice in TBST and incubated for 2 h with the HRP-conjugated secondary antibody, goat anti-mouse IgG (1:1000; Dingguo Biotech. Co., Beijing, China). After the membranes were washed in TBST, the signal was detected by the ECL system (Pierce, Thermo Scientific, USA). A western blotting of β-actin was performed in the same way, by using a mouse anti-actin antibody as the first antibody and a goat anti-mouse-HRP antibody (1:1000; Dingguo Biotech. Co., Beijing, China) as the second antibody. Each p53 band was normalized with respect to the corresponding β-actin band and the values were expressed as an intensity ratio.

### Immunohistochemistry for cell apoptosis

TUNEL (terminal deoxynucleotidyl transferase-mediated nick end labeling) staining was performed on melanoma tissues from mice. Apoptotic cells were detected using DAB in situ apoptosis detection kit according to the manufacturer's instructions. Stained sections were visualized under an optical microscope (Olympus, Japan).

### Statistical analysis

Data were expressed as mean ± SD. Results were analyzed by Student's t test in Fig. 2. One-way analysis of variance (ANOVA) followed by Tukey post hoc test was used in other studies. Differences were considered significant when *p* < 0.05.
